# Efficient Segmental Isotope Labeling of Integral Membrane Proteins for High-Resolution NMR Studies

**DOI:** 10.1021/jacs.4c03294

**Published:** 2024-05-24

**Authors:** Melina Daniilidis, Laura E. Sperl, Benedikt S. Müller, Antonia Babl, Franz Hagn

**Affiliations:** † Bavarian NMR Center, Department of Bioscience, School of Natural Sciences, Technical University of Munich, Ernst-Otto-Fischer-Str. 2, 85748 Garching, Germany; ‡ Institute of Structural Biology, Helmholtz Munich, Ingolstädter Landstr. 1, 85764 Neuherberg, Germany

## Abstract

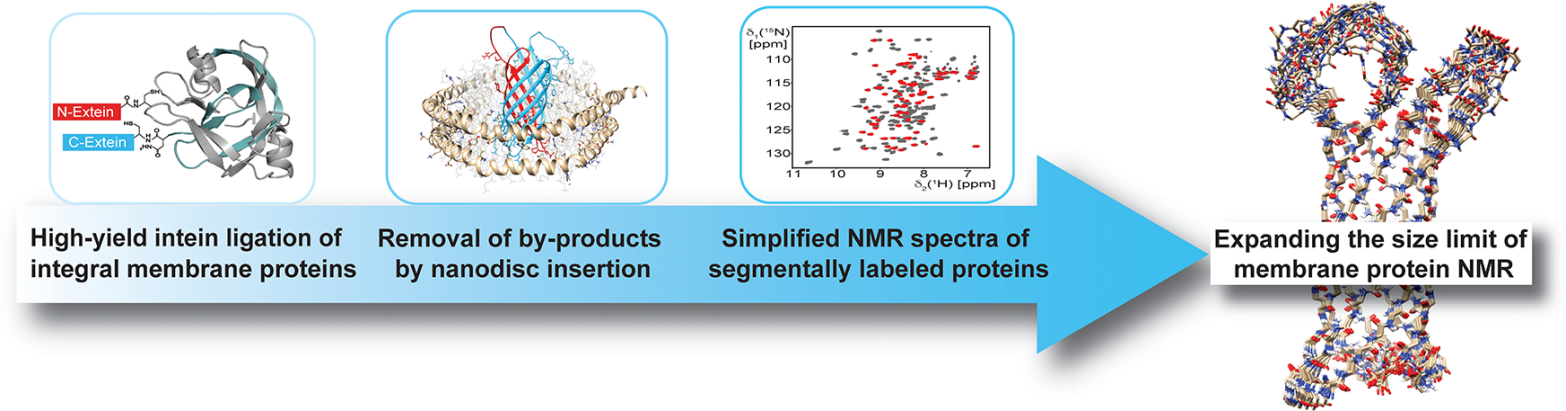

High-resolution structural NMR analyses of membrane proteins are challenging due to their large size, resulting in broad resonances and strong signal overlap. Among the isotope labeling methods that can remedy this situation, segmental isotope labeling is a suitable strategy to simplify NMR spectra and retain high-resolution structural information. However, protein ligation within integral membrane proteins is complicated since the hydrophobic protein fragments are insoluble, and the removal of ligation side-products is elaborate. Here, we show that a stabilized split-intein system can be used for rapid and high-yield protein trans-splicing of integral membrane proteins under denaturing conditions. This setup enables segmental isotope labeling experiments within folded protein domains for NMR studies. We show that high-quality NMR spectra of markedly reduced complexity can be obtained in detergent micelles and lipid nanodiscs. Of note, the nanodisc insertion step specifically selects for the ligated and correctly folded membrane protein and simultaneously removes ligation byproducts. Using this tailored workflow, we show that high-resolution NMR structure determination is strongly facilitated with just two segmentally isotope-labeled membrane protein samples. The presented method will be broadly applicable to structural and dynamical investigations of (membrane-) proteins and their complexes by solution and solid-state NMR but also other structural methods where segmental labeling is beneficial.

## Introduction

Membrane proteins are challenging systems for high-resolution structural analyses. These proteins are difficult to crystallize, and cryo-EM is particularly suited for large membrane protein systems.^1^ Small- to medium-sized membrane proteins can be investigated by solution- or solid-state NMR.^2^ However, adverse relaxation properties and limited long-term stability render NMR studies of larger membrane proteins very challenging. The use of high-level isotope-labeled protein and a suitable native-like membrane mimetic, such as lipid nanodiscs, can in part remedy this situation.^3,4^ Despite these important advancements, severe NMR signal overlap with larger proteins still markedly reduces spectral resolution and impedes the data analysis for resonance assignment and structure determination.^5^ This issue is particularly limiting for solid-state NMR experiments, where ^1^H line widths are markedly larger.^6^ Amino acid-selective isotope labeling^7^ has been used to simplify the NMR resonance assignment procedure. However, this approach requires the production of multiple samples which can be expensive regarding time and reagent costs. Segmental isotope labeling has turned out to be effective in simplifying NMR spectra.^8^ For segmental isotope labeling, various protein ligation methods have been reported, such as enzymatic approaches using trans peptidases,^9^ expressed protein ligation,^10,11^ and split-intein mediated trans-splicing.^12−14^ Typically, expressed protein ligation, or native chemical ligation,^15^ requires a C-terminal thioester and an N-terminal cysteine. The ligation yield can be further enhanced by using selenium cysteine at the ligation site.^16^ However, for expressed protein ligation, at least one fragment is typically of synthetic origin. A major advantage of intein-mediated trans-splicing is the direct use of recombinant protein fragments without the need for specific chemical modifications or synthetic educts for the ligation reaction.^17^ While trans-splicing of soluble (multi-) protein domains can be done in very good yields and thus turned out to be very useful for structural studies by NMR,^8^ the ligation within a compactly folded integral membrane protein remains difficult for various reasons, such as solubility, sample purity as well as ligation efficacy and product yield.^18^ Due to these obstacles, it was so far very difficult to produce sufficient amounts of segmentally isotope labeled membrane proteins by intein trans-splicing for high-resolution NMR studies.

Here, we used a highly stable DnaE split-intein system^19^ for high-yield protein trans splicing of integral membrane proteins. We showcase this method by performing segmental isotope labeling of an α-helical and a β-barrel integral membrane protein for high-resolution NMR studies. The obtained NMR spectra are markedly simplified, facilitating NMR spectral analysis and resonance assignment work. In addition, the insertion into lipid nanodiscs can serve as a quality control filter, rendering the time-consuming removal of ligation side products unnecessary. With this optimized workflow, NMR structure determination of integral membrane proteins is markedly facilitated, contributing to expanding the size limit of membrane proteins that are accessible to solution- and solid-state NMR. In addition, this workflow will be beneficial for other methods that rely on the selective integration of labels or chemical probes.

## Results

**Figure 1:**
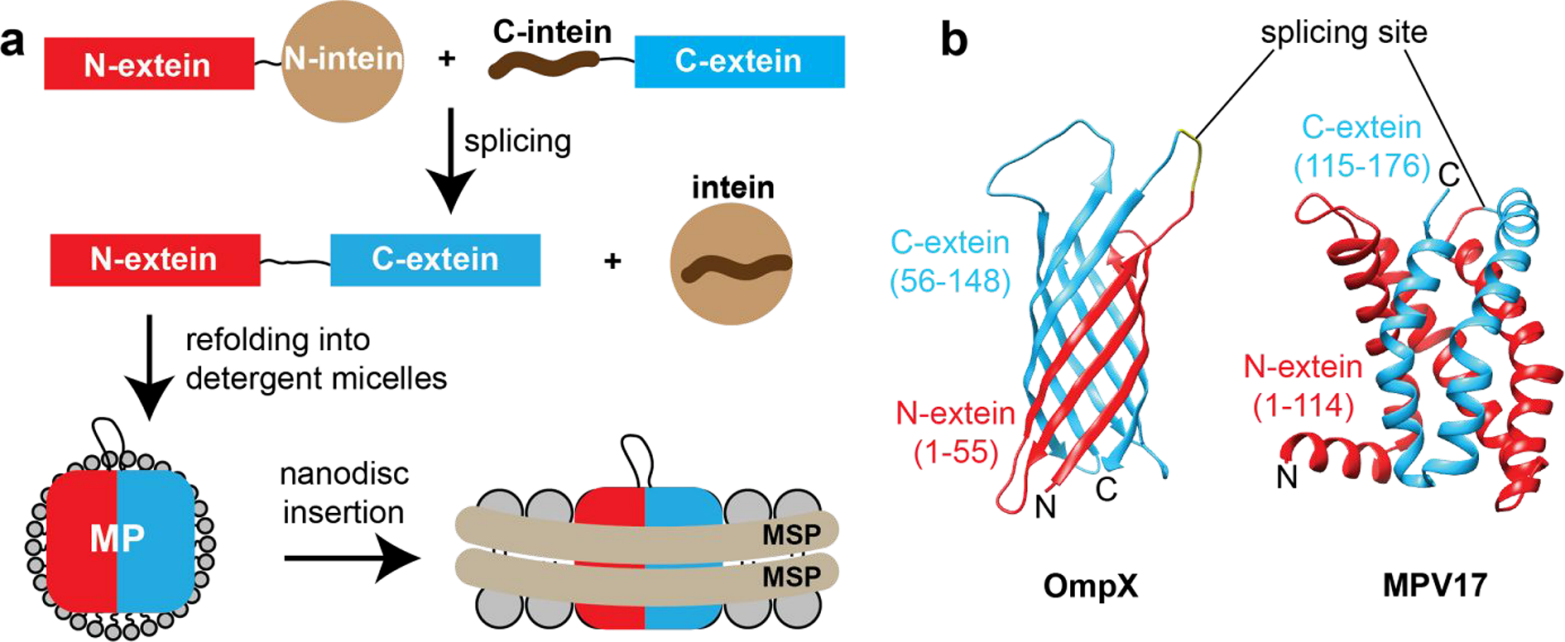
Intein-mediated protein trans-splicing for segmental isotope labeling of membrane proteins for NMR studies. (a) Strategy to obtain properly folded membrane proteins in detergent micelles and nanodiscs by protein trans-splicing. (b) Prototype membrane proteins OmpX (2m06.pdb^4^) and MPV17 (Alphafold^20^ model) with a β-barrel and α-helical topology, respectively. The N- and C-exteins and the splicing sites are indicated. MSP, membrane scaffold protein; MP, membrane protein.

**Figure 2:**
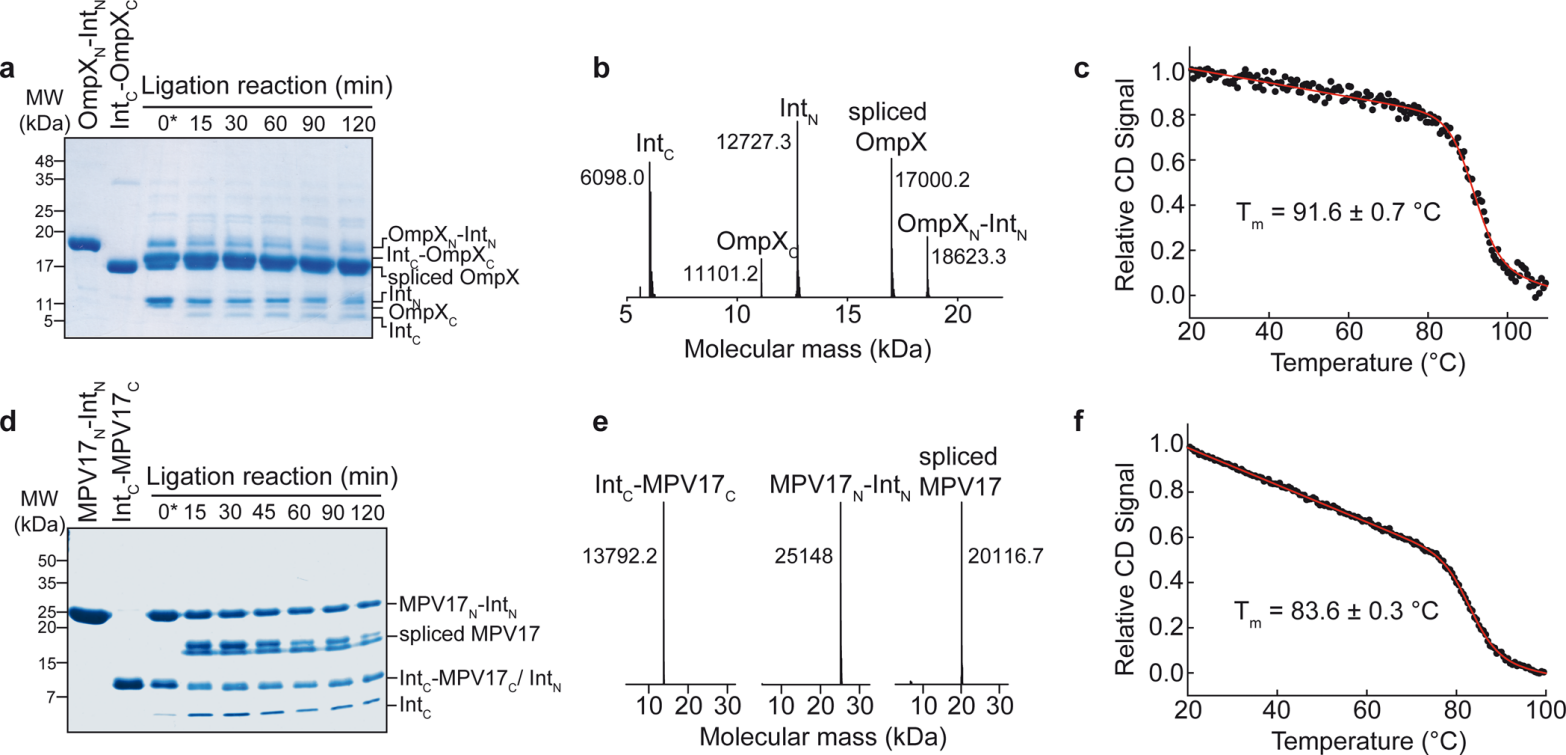
High-yield production of membrane proteins by intein-mediated protein trans-splicing. (a) SDS-PAGE of the splicing reaction of OmpX. (b) ESI-MS data of the splicing reaction indicating the generation of full-length OmpX. (c) Thermal stability of spliced OmpX refolded into DPC micelles. (d) same as in (a) but with the α-helical membrane protein MPV17. (e) ESI-MS data of the intein fragments and the spliced MPV17 product. (f) Same as in (c) but with MPV17 in DPC micelles. (a,d) 0* indicates the ∼10 s time point right after mixing the two OmpX or MPV17 fragments.

First, we designed suitable protein constructs for intein trans-splicing, where the N-terminal membrane protein fragment is fused to the N-intein and the C-terminal part to the C-intein (Figures 1a, S1, Supporting Information Materials and Methods section). Since the membrane protein fragments are predominantly insoluble in aqueous solution, the ligation reaction needs to be conducted under denaturing conditions, leading to a reduction in the acitivity of the intein. The Cfa intein used in this study^19^ is still very active at high urea or guanidine hydrochloride concentrations and is thus compatible with our ligation strategy. This setup allows for intein-mediated trans-splicing within a compactly folded membrane protein. The ligated full-length membrane protein can then be refolded into detergent micelles and, if desired, subsequently inserted into lipid bilayer nanodiscs of a suitable size.^4,21^ To demonstrate that this approach is feasible for membrane proteins of different secondary structures and topologies, we used the bacterial β-barrel membrane protein OmpX and the human α-helical inner mitochondrial membrane protein MPV17 as model systems (Figure 1b). The trans-splicing activity was reported to be dependent on the C-extein residues directly flanking the intein.^17^ To address this requirement, we here placed the splicing sites within loops to be able to incorporate additional residues that are beneficial for high splicing activity without perturbing the protein fold. If the structure of the membrane protein is not known, loop regions can be identified by secondary structure or AlphaFold^20^ predictions or using secondary chemical shift information from experimental NMR backbone resonance assignment data.^22^ Here, we incorporated the motif CFN at the C-extein positions 1–3, which is found in the native extein sequence of the naturally occurring split intein DnaE from *Nostoc punctiforme*.^23^ Furthermore, we added short linkers next to the exteins that have previously been shown to favor efficient splicing (Figure S1).^19,23^ If the length of a loop cannot be expanded, it is also possible to replace existing loop residues with this optimized amino acid stretch. In addition, it is recommended to evaluate the biophysical and functional properties of the desired protein construct as well as its refolding properties in advance using a full-length protein modified accordingly. For designing suitable OmpX constructs, we used the previously obtained NMR structure in lipid nanodiscs (2m06.pdb^4^). For MPV17, the AlphaFold^20^ model together with NMR backbone chemical shift information^24^ was used (Figures 1b, and S1). Each split-intein fusion protein was produced in *E. coli* and purified from inclusion bodies under denaturing conditions. The protein ligation reaction was performed in 6 M urea at 30 °C (Figure 2). With OmpX, the trans-splicing reaction was already close to completion after 15 to 30 min, with a maximum conversion of ∼75% determined after 120 min by SDS-PAGE (Figures 2a, and S2). The use of the Cfa intein led to a strongly increased yield and markedly reduced reaction times compared to a native split intein that has been previously used for the ligation of the bacterial outer membrane protein OmpF.^18^ Since the bands in the SDS-PAGE of the C-terminal Intein-OmpX construct (Int_C_-OmpX_C_) and the spliced full-length OmpX product almost completely overlapped, we used mass spectrometry to probe the mass (17 kDa) of the desired full-length OmpX product (Figures 2b, and S3).

Full-length OmpX obtained by intein trans-splicing (Figure S4a) shows identical thermal stability as wild-type OmpX, indicating that the slight sequence modifications required for efficient ligation did not perturb the protein structure (Figures 2c, and S4b). For the inner mitochondrial membrane protein MPV17, the splicing reaction was completed after 60 min, as probed by SDS-PAGE, resulting in a conversion of ∼50% after 120 min reaction time (Figures 2d, and S5), which was still enough to produce milligram quantities of the spliced protein. The spliced full-length MPV17 as well as the educts and the cleaved intein fragments were identified by mass spectrometry (Figure 2e). During the splicing reaction, full-length MPV17 precipitated in 6 M urea, whereas the educts remained soluble, which contributed to the high product yield and resulted in a high product purity. Since the soluble fraction was used for SDS-PAGE, the intensity of the band for full-length MPV17 consequently decreased with increasing reaction time (Figures 2d, and S6). As described above for OmpX, the introduction of a short linker for optimal ligation efficiency did not perturb the protein structure, as evident from a similar thermal melting point of intein-ligated MPV17 in DPC micelles compared to wild-type MPV17^24^ (Figure 2f).

Since some protocols utilize SDS for the solubilization of membrane proteins prior to refolding^25^ we wondered whether the Cfa-intein trans-splicing reaction can also be performed in 0.5% SDS, a typical concentration that is used for solubilization. As shown in Figure S7, product formation could not be observed, which can be rationalized by unfolding of the intein by this harsh detergent. However, this limitation does not prevent the solubilization of ligated membrane proteins in SDS for subsequent refolding experiments. To achieve this, the urea in the ligation reaction just needs to be removed by dialysis, followed by solubilization of the resulting protein precipitate in SDS. In contrast to intein trans-splicing, expressed protein ligation, not relying on the folding state of an intein, has been shown to be compatible with a harsh detergent environment.^10^

A common side product of intein trans-splicing reactions is the C-terminal extein without the C-intein fragment,^26^ together with unreacted educts. In our setup, we also observed the presence of the C-terminal OmpX fragment without the intein (OmpX_C_). His-tagged educts and inteins could be successfully removed by IMAC (Figure S8a). The OmpX_C_ fragment lacks a His-tag and was consequently copurified with the spliced OmpX product (Figure S8b). Finally, the removal of this unwanted fragment was achieved by elaborate size exclusion chromatography under denaturing conditions (Figure S9).

Next, we applied this tailored and optimized protocol to perform segmental isotope labeling of both membrane proteins. For this, we produced OmpX and MPV17 samples where each extein was either unlabeled (^1^H,^14^N) or isotope labeled (^2^H,^15^N) and visible in NMR (see Figure 1b). OmpX samples were inserted into size-optimized lipid nanodiscs^4,21,27^ containing a DMPC/DMPG (3:1) lipid blend (Figure S10). MPV17 forms large oligomers in lipids^24^ which prevented its insertion into lipid nanodiscs. Thus, the purification (Figure S11) and the NMR experiments were instead conducted in DPC micelles as previously described.^24^

**Figure 3:**
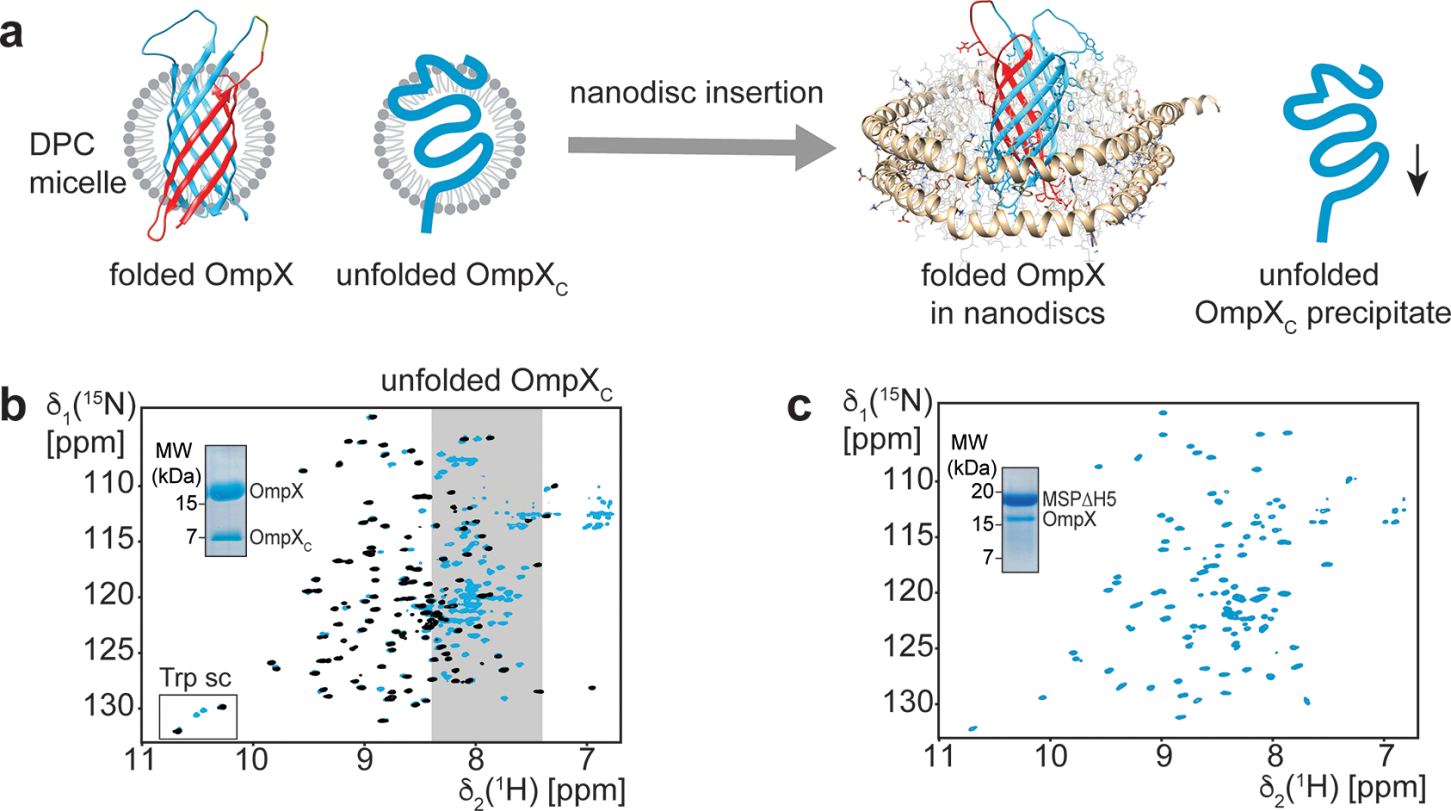
Removal of ligation side products by insertion into lipid nanodiscs. (a) After intein splicing and refolding, properly folded full-length and a misfolded OmpX_C_ fragment are copurifying in DPC detergent micelles, giving rise to a 2D-[^15^N,^1^H]-TROSY spectrum containing signals of both species (blue spectrum in panel b). The black spectrum in (b) is a reference with folded *U*-[^2^H,^15^N]-labeled OmpX in DPC micelles. Insertion into lipid nanodiscs selects for the properly folded species and efficiently removes the OmpX_C_ fragment, indicated by SDS-PAGE and a 2D-[^15^N,^1^H]-TROSY spectrum lacking signals in the unfolded region (c). NMR spectra were recorded at 318 K and at 950 MHz ^1^H frequency with a sample where the C-extein of OmpX (Figure 1b) is labeled with ^2^H and ^15^N (blue spectra). The N-extein is unlabeled and not visible in NMR. Trp sc: tryptophane NHε side chain signals.

To our positive surprise, the nanodisc assembly step with ligated OmpX served as a protein quality control filter since only compactly folded full-length protein inserted into the lipid environment of a nanodisc (Figures 3a, and S12). This approach allowed for skipping the elaborate size exclusion chromatography step under denaturing conditions (Figure S9) to remove OmpX_C_. NMR spectra of spliced OmpX containing ^2^H,^15^N-labeled OmpX_C_ in detergent micelles (Figure 3b) and lipid nanodiscs (Figure 3c) show that the undesired set of signals corresponding to the unfolded OmpX_C_ fragment in DPC micelles are completely absent in lipid nanodiscs. Thus, the nanodisc insertion procedure can be considered a general and convenient way for efficient product purification, rendering more elaborate purification strategies obsolete.

**Figure 4:**
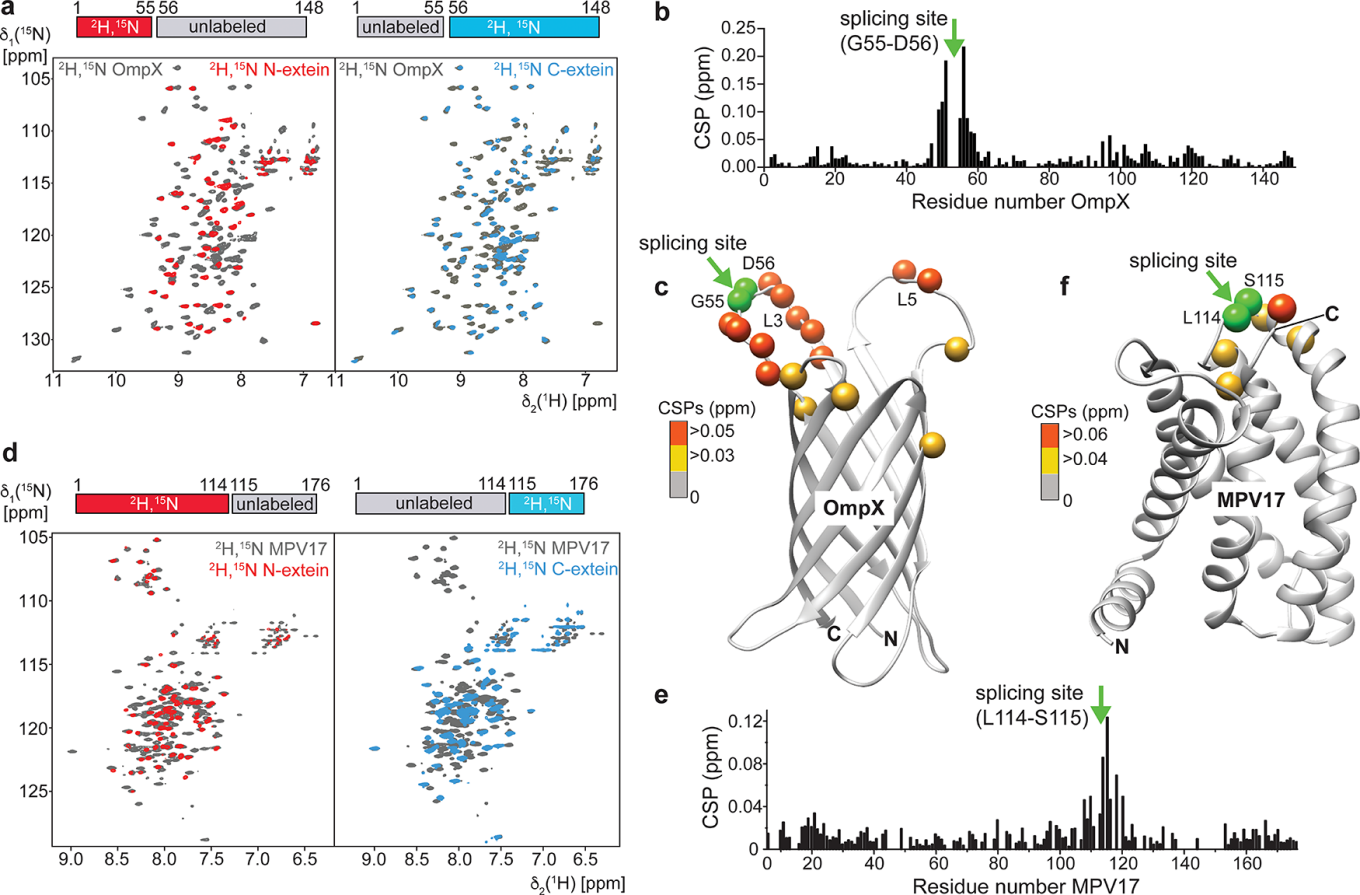
2D-NMR analysis of the segmentally isotope-labeled integral membrane proteins OmpX and MPV17. (a) 2D-[^15^N,^1^H]-TROSY spectra of uniformly labeled OmpX (gray) and segmentally labeled OmpX (red and blue). (b) Chemical shift perturbations (CSPs) within OmpX calculated from the spectra shown in (a). (c) CSPs mapped onto the structure of OmpX (2m06.pdb^4^). The splicing site is indicated by the two green spheres. (d–f) Same as in (a–c) but with segmentally isotope labeled MPV17 in DPC micelles. The structural model of MPV17 was obtained with AlphaFold.^20^

With this optimized ligation and purification workflow, we next recorded 2D-[^15^N,^1^H]-TROSY spectra of segmentally isotope-labeled OmpX (*vide supra*) in lipid nanodiscs (Figure 4a) and compared them with spectra of uniformly ^2^H,^15^N-labeled OmpX. In these spectra, the signals of segmentally labeled OmpX overlaid almost perfectly with the corresponding signals of uniformly labeled OmpX. Slight chemical shift perturbations were only observed in the region around the splicing site and the neighboring loop region (Figure 4b,c).

Overall, the complexity of the spectra is markedly reduced due to a lower number of NMR signals, which is most pronounced in the central region of the spectrum where unfolded protein stretches usually lead to heavy signal overlap. NMR spectra of similar quality were obtained with segmentally labeled MPV17 in detergent micelles (Figure 4d). For this α-helical membrane protein, where signal dispersion is less pronounced leading to strong overlap, the benefit of segmental labeling is even more apparent. In line with the very similar thermal stability data (Figure 2f), the NMR spectra are only slightly perturbed around the splicing site (Figure 4e,f), indicating that the structure of MPV17 is not altered.

**Figure 5:**
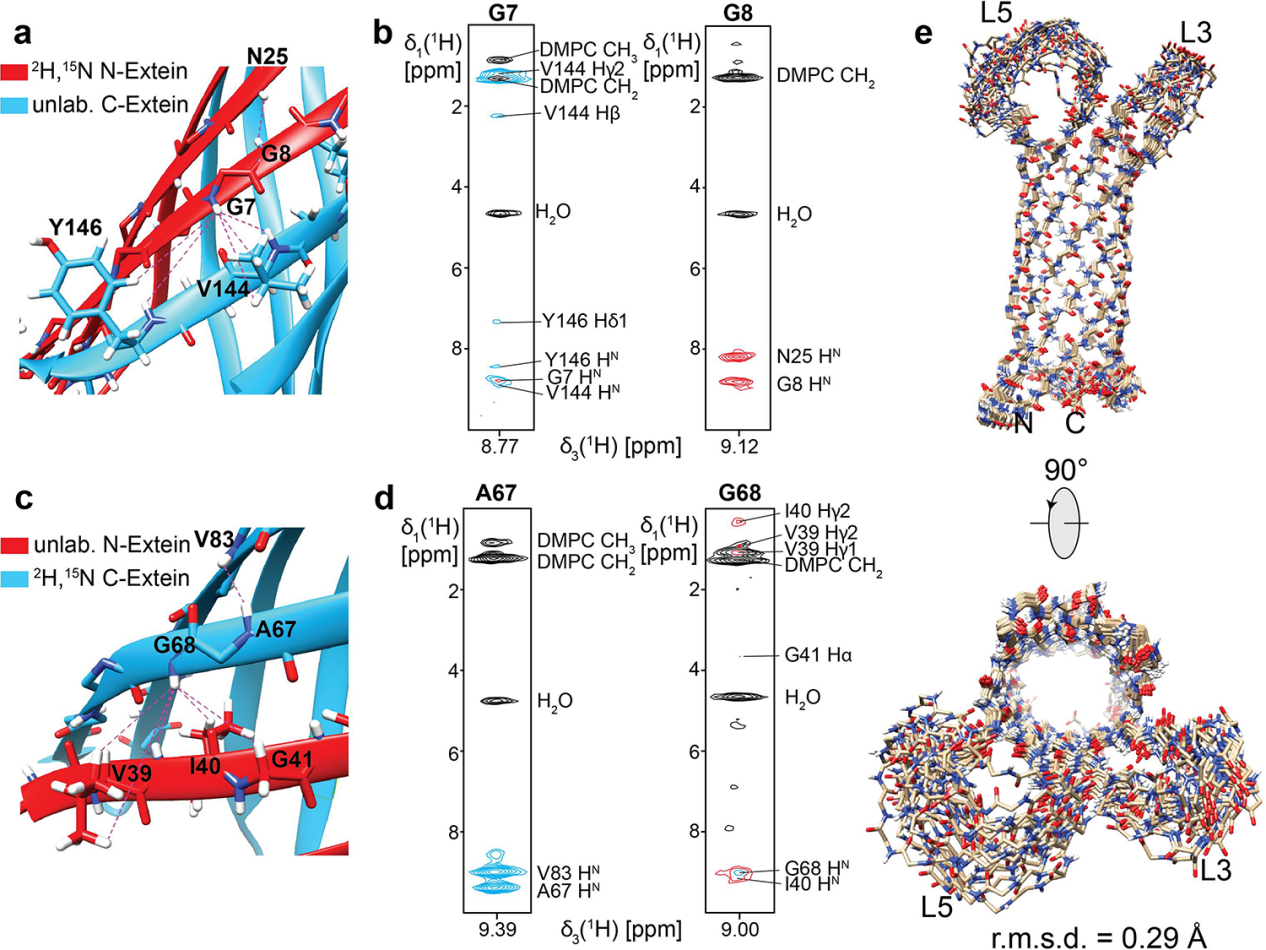
Segmental isotope labeling of membrane proteins facilitates NMR structure determination. (a) Structural interface between ^2^H,^15^N-labeled OmpX-N-extein and unlabeled OmpX-C-extein with observed NOE distance restraints (shown in (b)) within the labeled or to the unlabeled part. (c) and (d), same as in (a) and (b) but with an inverse isotope labeling pattern, i.e., ^2^H,^15^N-labeled OmpX-C-extein and unlabeled OmpX-N-extein. (e) 20 lowest energy structures obtained with the two segmentally labeled OmpX samples in lipid nanodiscs, showing an root mean square deviation (rmsd) of backbone atoms in ordered secondary structure elements of 0.29 Å. The assignment of NOE contacts was facilitated by the lower complexity and signal overlap in the individual spectra.

Due to the simplification of the NMR spectra of segmentally isotope-labeled proteins, NMR structure determination is considered to be markedly facilitated.^13,14^ Typically, this strategy requires the production of two orthogonally labeled samples, e.g., only one segment is labeled with NMR-active nuclei with the other one only ^1^H labeled and *vice versa*. Here, we used a ^2^H,^15^N versus ^1^H,^14^N labeling pattern (see Figure 4a), which is sufficient for the structure determination of smaller β-barrel membrane proteins^4^ using backbone NOE contacts. However, any isotope labeling pattern on each side can be used if necessary, including selective ^13^C methyl labeling^275^ in both fragments to enable the detection of specific contacts within larger integral membrane proteins. For extracting distance restraints, we recorded 3D-^15^N-edited-TROSY-[^1^H,^1^H]-NOESY NMR experiments with the two segmentally labeled OmpX samples in lipid nanodiscs. As expected, we could observe NOE-connections within the isotope-labeled segment in each sample (Figure 5a–d), e.g., G8-N25 or A67-V83 amide proton NOEs.

In addition, at the edges of each isotope-labeled segment, NOE contacts are visible between an ^15^N-isotope-labeled amide and any proton in the adjacent unlabeled (^1^H) β-stand, e.g., G7-Y146, G7-V144 or G68-I40, G68-V39. This pattern further facilitated the assignment of NOE contacts since not only the amide of a cross-β-stand residue is detected but also its side chain protons. Moreover, additional NOE restraints can be used for structure determination, providing additional structural information in these regions.

With this strategy, we could collect 180 NOE distance restraints to determine a high-resolution structure of OmpX in lipid nanodiscs (Figure 5e) with a backbone atom root mean squared deviation (rmsd) of 0.29 Å (Table 1), which is slightly better than our previous structure based on backbone amide NOEs only.^4^ It can be further envisioned that this approach will be beneficial to unambiguously probe distances across β-barrels or α-helical bundles between selectively labeled protein fragments using orthogonal isotope labeling patterns in larger membrane proteins. Side-chain methyl group spectra typically show heavy overlap but are essential to determine accurate tertiary structures, even for β-barrel membrane proteins.^28^

**Table 1:** Structural Statistics of Segmentally Labeled OmpX in Phospholipid Nanodiscs^a^

structural information	
NOEs (amide and side-chain contacts)	180
Hydrogen bond restraints	77
Dihedral angle restraints (TALOS^29^)	291
Backbone rmsd in β-strands (Å)^b^	0.29 ± 0.05
Backbone rmsd for all residues (Å)	1.12 ± 0.29
Ramachandran map analysis^c^	
Most favored regions	93.4%
Additionally allowed regions	4.1%
Generously allowed regions	2.5%
Disallowed regions	0.0%
Deviations from restraints and idealized geometry	
Distance restraints (Å)	0.11 ± 0.03
Dihedral angle restraints (°)	0.19 ± 0.02
Bonds (Å)	0.0024 ± 0.00008
Angles (°)	0.66 ± 0.01
Impropers (°)	1.91 ± 0.13

a Analysis of the 20 lowest-energy structures.

b Only ordered secondary structure elements were used for structural superimposition: 3–14, 20–30, 38–48, 60–71, 78–90, 104–115, 122–132, 135–147; rmsd values are calculated relative to a nonminimized average structure of each ensemble.

c Ramachandran analysis with PROCHECK-NMR^30^ was performed on the lowest-energy structure.

## Discussion

This study provides the first example where high-resolution NMR structure determination was conducted with a segmental isotope labeled integral membrane protein. Segmental isotope labeling for NMR has been used for decades but is limited to soluble proteins or, e.g., attaching unfolded tails to membrane-associated domains.^14^ Here, we use the exceptional properties of the Cfa intein^19^ to perform protein ligation by intein-splicing within an integral membrane protein. In comparison to a previous study,^18^ the exceptionally fast reaction rate and high yield of the protein trans-splicing with the Cfa intein under denaturing conditions enables the production of milligram quantities of segmental isotope-labeled membrane proteins and renders this approach feasible for a wide range of membrane protein systems where refolding is possible. To obtain optimal protein ligation yields we here used naturally occurring sequence elements between the inteins and the exteins. This strategy requires the positioning of the splicing sites in loop regions of the membrane protein. However, recent improvements of the Cfa intein^31^ render traceless splicing more efficient with markedly reduced sequence requirements at the splicing site. If a particular membrane protein system requires segmental isotope labeling of an internal fragment, orthogonal split inteins can in principle be used to ligate three segments.^32^ However, since every additional ligation step reduces the overall protein yield, the design of fast and efficient orthogonal split inteins that are active under denaturing conditions will be necessary to make this approach usable for NMR sample preparation.

NMR investigations of membrane proteins require not only high-field instrumentation and optimized experimental setup but also cutting-edge biochemical sample production methods. The use of any isotope-labeling strategy that simplifies the NMR spectral signature and renders the NMR resonance assignment procedure less elaborate is essential for enabling investigations of the structure, interactions, and dynamics of membrane proteins of increasing complexity. An important class of integral membrane proteins are G-protein coupled receptors (GPCRs), where in some cases the production in *E. coli* and refolding was shown to be possible^33^ although high-level isotope labeling and NMR resonance assignments are still challenging.^34^ Thus, our approach might be used to specifically visualize functional elements in such larger membrane proteins to probe their conformational states and interactions with small molecules and partner proteins.

While we here used solution-state NMR, we anticipate that our method will also be highly beneficial for solid-state NMR studies of membrane proteins. At ultra-fast magic angle spinning conditions and at ultra-high magnetic field, solid-state NMR is in principle size-independent,^6,35^ enabling investigations of very large systems. However, the increasing number of resonances in large proteins impedes the spectral analysis due to heavy signal overlap. The ability to segmentally label selected regions of interest will thus also be essential for structural studies of larger membrane proteins by solid-state NMR. In addition to NMR studies, the presented approach can be further used to selectively attach spin labels to desired protein segments within a folded (membrane) protein domain for electron paramagnetic resonance (EPR) spectroscopy. This procedure renders mutagenesis of surface-exposed reactive amino acid residues in parts of the protein where no labeling is desired obsolete.^36^ Furthermore, segmental isotope labeling of membrane proteins might be useful for small-angle neutron scattering (SANS) experiments,^37^ where contrast matching can be employed to selectively observe conformational changes of a particular structural element within a folded membrane protein. Hence, we believe that the presented method will be broadly applicable to the investigation of the structure, dynamics, and function of integral membrane proteins and other large protein systems.

## Conclusions

We showed that intein-based trans-splicing can be conducted at high yields with integral membrane proteins. This setup opens the possibility for segmental isotope labeling for high-resolution NMR studies, where signal overlap is a main issue that prevents a detailed analysis of larger and more challenging systems. We show that lipid nanodisc insertion provides an efficient platform for selecting the correctly ligated and properly folded membrane protein species. This workflow facilitates the high-resolution structure determination of membrane proteins in a native lipid environment and will be a versatile tool to selectively study the structure and dynamics of functionally important parts of membrane proteins by NMR and other structural methods.

## Supporting Information Available

Additional experimental details, materials, and methods, including figures on split-intein fusion protein construct design, reaction yields, mass spectrometry, purification of the protein constructs and ligation products, and Supporting Information references (PDF)

## Abbreviations

DPC: dodecylphosphocholine
MSP: membrane scaffold protein
MS: mass spectrometry
NMR: nuclear magnetic resonance
SEC: size exclusion chromatography
IMAC: immobilized metal ion affinity chromatography
SDS: sodium dodecyl sulfate
SDS-PAGE: sodium dodecyl sulfate-polyacrylamide gel electrophoresis
