# Supplementary material for: Efficient Segmental Isotope Labeling of Integral Membrane Proteins for High-Resolution NMR Studies
Source: J Am Chem Soc. 2024 May 24;146(22):15403–10. doi: 10.1021/jacs.4c03294 (PMC11157531; doi:10.1021/jacs.4c03294)
Supplement: Supplementary file 1 — ja4c03294_si_001.pdf [file ja4c03294_si_001.pdf]

Supporting Information to:

Efficient segmental isotope labeling of integral membrane proteins for high-resolution NMR studies

Melina Daniilidis[1], Laura E. Sperl[1,†], Benedikt S. Müller[1], Antonia Babl[1] & Franz Hagn[1,2,*]

[1]Bavarian NMR Center, Department of Bioscience, School of Natural Sciences, Technical University of Munich, Ernst-Otto-Fischer-Str. 2, 85748 Garching, Germany; [2]Institute of Structural Biology, Helmholtz Munich, Ingolstädter Landstr. 1, 85764 Neuherberg, Germany

* Correspondence to: franz.hagn@tum.de

Supporting Information Materials and Methods

Construct design. We designed consensus fast DnaE (Cfa) split-intein[1] fusion protein constructs with two different membrane proteins, β-barrel protein OmpX and α-helical MPV17. For OmpX, the N- and C-terminal intein fusion constructs were: OmpXΔS_{1-55}-G-Int_N(Cys1-Pro101)-LE-His_6 and MSYY-His_6-DYDIPTTA-Int_C(MV_{102}-ASN_{136})-CFN-SGG-$OmpX_{56-148}$. For MPV17, the constructs were: $MPV17_{1-114}$-SSGG-Int_N-LE-His_6 and MSYY-His_6-DYDIPTTA-Int_C-CFN-$MPV17_{115-176}$. For this study we used a well-behaving MPV17 variant where intrinsic cysteine residues were removed[2].

OmpX constructs

$OmpX_N$-Int_N:

MATSTVTGGYAQSDAQGQMNKMGGFNLKYRYEEDNSPLGVIGSFTYTEKSRTASSGGCLSYDTEILTVE
YGFLPIGKIVEERIECTVYTVDKNGFVYTQPIAQWHNRGEQEVFEYCLEDGSIIRATKDHKFMTTDGQM
LPIDEIFERGLDLKQVDGLPLEHHHHHH

Int_C-$OmpX_C$:

MSYYHHHHHHDYDIPTTAMVKIISRKSLGTQNVYDIGVEKDHNFLLKNGLVASNCFNSGGDYNKNQYY
GITAGPAYRINDWASIYGVVGVGYGKFQTTEYPTYKHDTSDYGFSYGAGLQFNPMENVALDFSYEQSRI
RSVDVGTWIAGVGYRF

MPV17 constructs

$MPV17_N$-Int_N:

MALWRAYQRALAAHPWKVQVLTAGSLMGLGDIISQQLVERRGLQEHQRGRTLTMVSLGAGFVGPVVG
GWYKVLDRFIPGTTKVDALKKMLLDQGGFAPAFLGAFLPLVGALNGLSSGGCLSYDTEILTVEYGFLPIG
KIVEERIECTVYTVDKNGFVYTQPIAQWHNRGEQEVFEYCLEDGSIIRATKDHKFMTTDGQMLPIDEIFE
RGLDLKQVDGLPLEHHHHHH

Int_C-MPV17$_C$:

MSYYHHHHHHDYDIPTTAMVKIISRKSLGTQNVYDIGVEKDHNFLLKNGLVASNCFNSAQDNWAKLQ
RDYPDALITNYYLWPAVQLANFYLVPLHYRLAVVQAVAVIWNSYLSWKAHRL

Protein production. The production of OmpX$_N$-Int$_N$, Int$_C$-OmpX$_C$ and OmpXΔS was carried out by transforming *E. coli* BL21(DE3) cells with OmpX$_N$-Int$_N$-pET25b, Int$_C$-OmpX$_C$-pET25b and OmpXΔS-pET11a[3] respectively. Protein expression was induced with 1 mM IPTG and cells were grown for 5 hours as described previously[3]. The production of MSPΔH5 was done according to previous protocols[3, 4]. The split-intein constructs of MPV17 were also produced in inclusion bodies in *E. coli* BL21(DE3) as described previously [2]. For ^{2}H,^{15}N-labeled protein, cells were grown in M9 media in 99% D_2O supplemented with 1 g/L $^{15}NH_4Cl$.

Protein purification. OmpXΔS purification and refolding were carried out according to existing protocols[3, 5]. The purification of OmpX$_N$-Int$_N$ and Int$_C$-OmpX$_C$ was conducted by resuspending the cells in 50 mM Tris-HCl pH 8.0, 300 mM NaCl, 1 mM EDTA, 10 mM BME and 1% Triton X-100 (Lysis Buffer) + 1 mM PMSF. Cells were then incubated with lysozyme for 30 min and sonicated. The lysate was incubated for 30 min with 100 U of DN*ase*I and 5 mM $MgCl_2$. The solution was then centrifuged at 4 °C for 30 min at 40 000 *g* and the pellet was resuspended in lysis buffer and centrifuged as above. This was repeated twice using the lysis buffer without Triton X-100. The pellet was then solubilized in 6 M GdmCl, 50 mM Tris-HCl pH 8.0, 100 mM NaCl and 5 mM BME. After centrifuging again at 4 °C for 30 min at 40 000 *g*, the supernatant was applied to a gravity flow Ni^{2+}-NTA column. The column was washed with 2 CV of the GdmCl buffer and protein then eluted using 6 CV of GdmCl buffer containing 500 mM imidazole. The protein was then further dialyzed to 20 mM Tris-HCl pH 8.0, 50 mM NaCl and 5 mM BME. The precipitated Int$_C$-OmpX$_C$ was solubilized in 2.5 mL GdmCl buffer. OmpX$_N$-Int$_N$ only precipitated in small quantities, thus the protein was mainly found in the soluble fraction which was further used and concentrated to a volume of 2.5 mL using Amicon (Millipore) centrifugal devices (10kDa MWCO). The MPV17 split-intein protein pellets were purified from inclusion bodies as described previously[2]. In brief, the protein pellet was dissolved in 6 M GdmCl buffer (50 mM Tris-HCl pH 8.0, 6 M GdmCl, 5 mM BME) and purified with a gravity flow Ni^{2+}-NTA column. The pure protein was precipitated by dialysis for further use in the protein splicing reaction.

Splicing reaction and purification. The splicing reaction was conducted as described previously by Steven *et al.*[1] OmpX$_N$-Int$_N$ and Int$_C$-OmpX$_C$ were each (2.5 mL) applied to NAP-25 columns (GE Healthcare), equilibrated with 6 M urea, 100 mM NaPi pH 7.2, 150 mM NaCl, 1 mM EDTA (splicing buffer). The proteins were then eluted using 3.5 mL of splicing buffer. The subsequent splicing assay was then conducted with a protein concentration of 100 μM of each OmpX$_N$-Int$_N$ and Int$_C$-OmpX$_C$ (1:1 molar ratio) and 2 mM TCEP. The reaction was incubated for 2 h at 30 °C while shaking at 300 rpm. Afterwards, the reaction was stopped using 4% (v/v) trifluoroacetic acid (TFA) in a 3:1 volumetric ratio (3 parts reaction mix, 1 part TFA). For splicing in 0.5% sodium dodecyl sulfate (SDS), Int$_C$-OmpX$_C$ was precipitated by dialysis in splicing buffer without urea and solubilized in the same buffer containing 0.5% SDS instead of urea. OmpX$_N$-Int$_N$ was also dialyzed against splicing buffer without urea and thereafter SDS was added to a final concentration of 0.5%. The intein splicing reaction was then carried out at 37 °C, otherwise equally to the approach in 6 M urea. To determine reaction rates and yields, samples were taken every 15 to 30 min

during the splicing reaction and applied to SDS-PAGE. The intensity decrease of the $OmpX_N$-Int_N band was determined using ImageJ[6] and plotted against the reaction time. After the splicing was stopped, the reaction was dialyzed against 50 mM Tris-HCl pH 8.0, 100 mM NaCl and 5 mM BME. The resulting precipitate was dissolved in 8 M urea, 50 mM Tris-HCl pH 8.0, 300 mM NaCl and 12 mM BME and then applied to a gravity flow Ni^{2+}-NTA column equilibrated in the same buffer. The column was washed with 2 CV urea buffer and His-tagged proteins then eluted using the urea buffer + 500 mM imidazole. Flow-through and wash fractions that contained the spliced product were collected and then dialyzed against 20 mM Tris-HCl pH 8.0, 25 mM NaCl, 5 mM DTT and 1 mM EDTA. The C-terminal fragment ($OmpX_C$) could either be removed from the spliced product by incorporating the refolded protein into nanodiscs or by purifying it with size-exclusion chromatography. In this case, the precipitated protein was solubilized in 3 mL of 6 M urea, 50 mM Tris-HCl pH 8.0, 150 mM NaCl, 5 mM DTT and then applied to a HiLoad 16/600 Superdex 75 pg size exclusion column (Cytiva). Fractions were analysed using SDS-PAGE and then pooled and dialyzed against 20 mM Tris-HCl pH 8.0, 25 mM NaCl, 1 mM EDTA, 5 mM DTT. Clean, precipitated protein was solubilized to a concentration of 5 mg/mL in 6 M GdmCl, 50 mM Tris-HCl pH 8.0, 100 mM NaCl and 5 mM BME.

The precipitates of the two MPV17 split-intein constructs were dissolved in splicing buffer (100 mM NaPi pH 7.0, 150 mM NaCl, 1 mM EDTA, 6 M urea, 10 mM DTT), followed by centrifugation to remove insoluble material. Equal amounts (50-100 µM each) of the Int_N and Int_C constructs were mixed and stirred at 30 °C for 2 h. The formation of full-length MPV17 lead to its precipitation in 6 M urea, whereas the precursor proteins were soluble under these conditions. Splicing was terminated by adding two reaction volumes of splicing buffer without urea and one reaction volume of 4 % (v/v) TFA on ice, leading to a urea concentration of 1.5 M and complete precipitation of the full-length splicing product. After centrifugation, the pellet was washed twice in splicing buffer without urea. To remove residual His_6-tagged precursor proteins, gravity flow Ni^{2+}-NTA chromatography was performed in equilibration buffer (50 mM Tris-HCl pH 8.0, 6 M GdmCl, 5 mM BME) where the product was collected in the flow-through and wash fractions (+ 10 mM imidazole). Subsequent dialysis against denaturant-free buffer led to protein precipitation, which was collected by centrifugation and used for the subsequent refolding step.

Refolding. Refolding[3, 5] of OmpX-ΔS and spliced OmpX (either with or without the fragment $OmpX_C$) was done at 4 °C by dropwise dilution of the protein into a 10-fold volume of buffer containing 50 mM Tris-HCl pH 8.5, 5 mM EDTA, 0.5 % DPC and 500 mM arginine while stirring for 3 more hours at 600 rpm after total addition of the protein. Afterwards, the solution was dialyzed against 20 mM Tris-HCl pH 8.0, 100 mM NaCl, 1 mM EDTA and 2 mM DTT. For MPV17, the pellet of the spliced protein was dissolved in 6 M GdmCl buffer, refolded dropwise into a 10-fold excess of refolding buffer (20 mM NaPi pH 7, 50 mM NaCl, 1 mM EDTA, 0.5 % DPC) and further purified by size exclusion chromatography, as described previously[2].

Reconstitution into lipid nanodiscs. Nanodisc formation[4] with OmpX and spliced OmpX was done with a protein:MSP:lipid ratio of 1:4:40 (50 µM Protein in DPC, 200 µM MSP1D1ΔH5, 8 mM DMPC:DMPG=3:1). The mixture was incubated at RT for 1 h and subsequently the detergent was removed by the addition of 1 g Biobeads-SM2 (Bio-Rad) per mL of assembly and gentle shaking at RT for 1.5 h. Assembled nanodiscs were further purified on a HiLoad 16/600 Superdex 200 pg size exclusion column (Cytiva) equilibrated with 20 mM NaPi pH 6.5, 50 mM NaCl, 1 mM EDTA

and 5 mM DTT and then concentrated to ~ 1 mM using Amicon (Millipore) centrifugal devices (10 kDa MWCO).

Electrospray ionization mass spectrometry (ESI-MS). Products of the splicing reaction were analyzed using an LCQ-Fleet system (Thermo Fisher Scientific) equipped with a 3D ion trap and ESI device. The MS instrument was connected to an UltiMate 3000 high-performance liquid chromatography (HPLC) system (Thermo UltiMate 3000, Thermo Fisher Scientific) with a 10 × 2.1 mm MSPac™ DS-10 MS, 5 µm column (Thermo Fisher Scientific).

Circular dichroism (CD) spectroscopy. CD measurements were performed on a Jasco J-715 spectropolarimeter with a 1 mm pathlength quartz cuvette. The spectra were measured at 20°C. Thermal unfolding was measured by monitoring the ellipticity at 222 nm for MPV17 and at 215 nm for OmpX, between 20 °C and 100°C or 120°C respectively, while heating with 1°C/min. Data were fitted to a custom Boltzmann equation for thermal unfolding[7]. Measurements were recorded in the respective refolding buffer for each protein.

NMR spectroscopy. 2D-[^{15}N,^{1}H]-TROSY-HSQC NMR experiments were performed at 310 K for MPV17 and at 318 K for OmpX on Bruker Avance III spectrometers operating at 900 and 950 MHz proton frequency, respectively, equipped with cryogenic probes and controlled by Topspin 3.5 (Bruker Biospin). The spectra were processed with Topspin 3.5 (Bruker Biospin) and the data analyzed with NMRFAM-Sparky[8]. Chemical shift perturbations were calculated as ^{1}H,^{15}N-averaged values, as described in Ref.[9] using the perturbation analysis module in NMRFAM-Sparky[8]. NMR-based structure determination of 1 mM segmentally isotope labeled OmpX in lipid nanodiscs (see above) was done as described recently[3, 10]. NOE distance restraints were extracted from 3D-^{15}N-edited-TROSY-[^{1}H,^{1}H]-NOESY NMR experiments recorded with two orthogonally labeled OmpX samples, i.e. one extein ^{2}H,^{15}N and the other extein ^{1}H,^{14}N labeled, and *vice versa*. After initial rounds of NOE-based structure calculation with Xplor-NIH[11] using standard protocols, we implemented hydrogen bond restraints for amide moieties that showed NOE contacts that are consistent with the existence of a hydrogen bond between the antiparallel b-strands of OmpX and where no NOE peak to the water resonance (~4.7 ppm ^{1}H) was observed. A final analysis of the backbone angles was performed with PROCHECK-NMR[12].

Supporting Information Figures

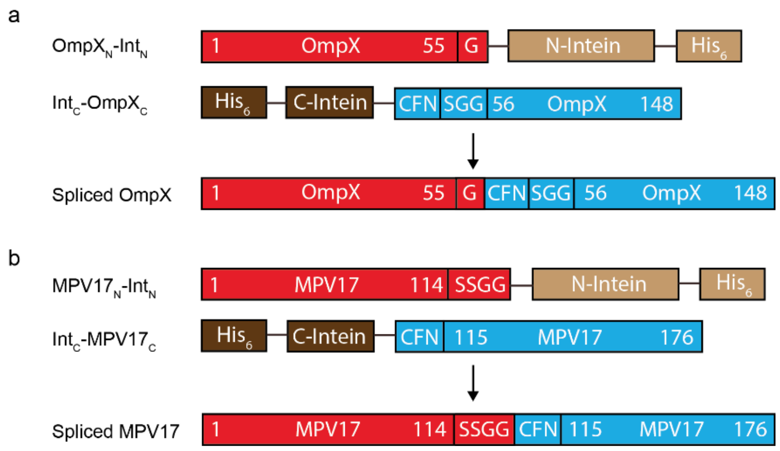

Supporting Information Figure 1. Design of splicing constructs. Protein sequences designed for splicing of (a) OmpX and (b) MPV17 with respective splicing products. Besides the glycine and serine linkers (G, (S)SGG), the canonical tripeptide CFN, which is important for efficient trans-splicing, is also indicated in the extein sequence.

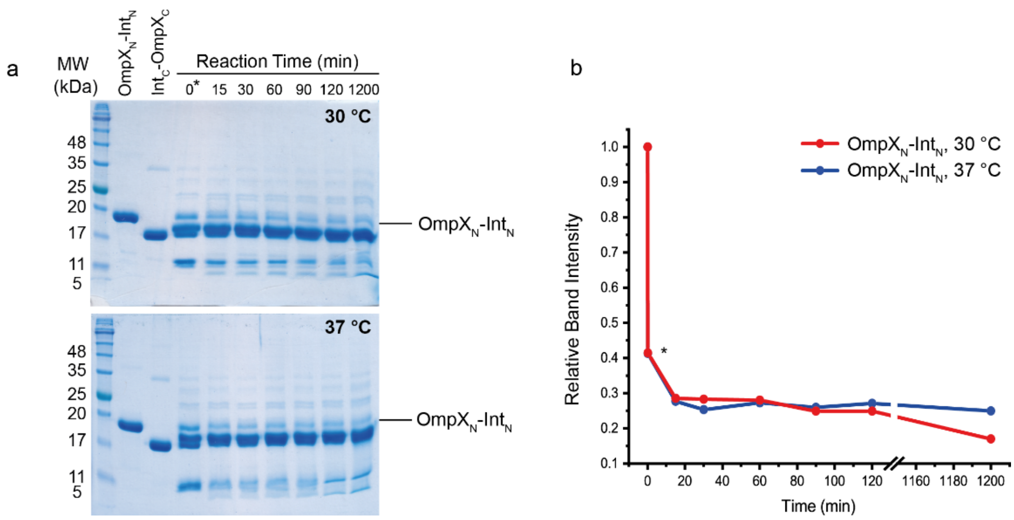

Supporting Information Figure 2. Reaction rate of OmpX splicing. Splicing was conducted in 6 M urea at 30 °C and 37 °C respectively, while SDS-PAGE samples were taken every 15 to 30 min and after 20 h (a). Relative band intensities of the educt OmpX$_N$-Int$_N$ were determined, and the reduction of band intensity plotted against the reaction time (b). For both temperatures most of the conversion was achieved after 15-30 min and yielded ligation rates of ~75% after 120 min. * The sample at time point zero was taken approximately 10 sec after mixing OmpX$_N$-Int$_N$ and Int$_C$-OmpX$_C$. Due to the high efficiency of the Cfa intein, 58% conversion was already reached at this point. Thus, values were calculated relative to the educt band of OmpX$_N$-Int$_N$.

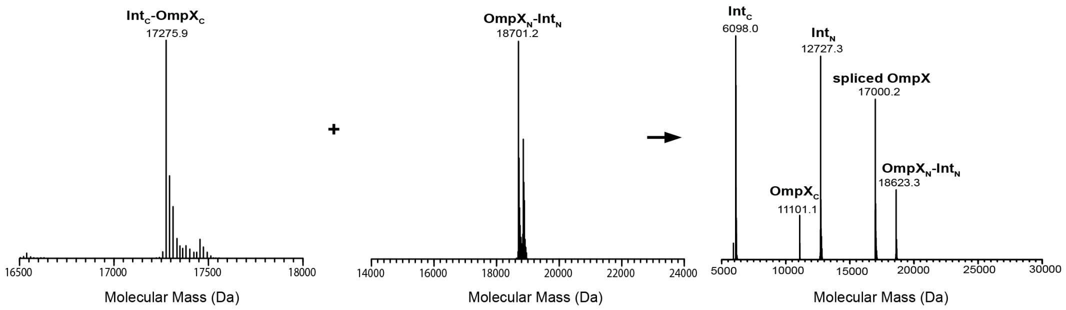

Supporting Information Figure 3. Electrospray-ionization mass-spectrometry (ESI-MS) data of splicing reaction. Molecular masses of the educts (Int$_C$-OmpX$_C$ and OmpX$_N$-Int$_N$) before the intein-splicing reaction (left) and after 120 min of splicing (right) were detected. Besides spliced OmpX, excised Int$_C$ and Int$_N$, as well as the educt OmpX$_N$-Int$_N$ could be identified after splicing. Int$_C$-OmpX$_C$ was not apparent in the MS spectrum. OmpX$_C$, a fragment forming due to the early cyclization of the catalytically active asparagine at the splice site, could however be detected.

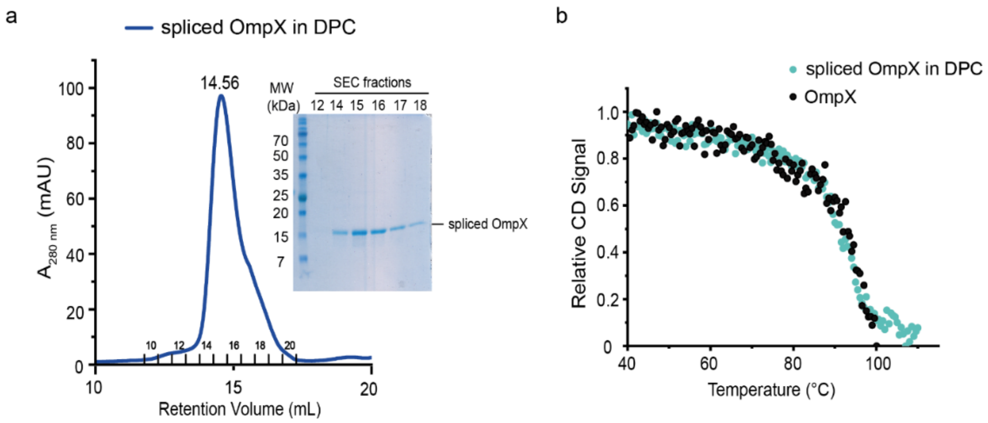

Supporting Information Figure 4. Thermal stability of purified spliced OmpX in DPC micelles. After purifying spliced OmpX by size-exclusion chromatography (SEC) in 6 M urea, pure protein was refolded in n-Dodecyl-phosphocholine (DPC) and applied to another SEC (a). Respective fractions are indicated in the adjacent SDS-PAGE. The thermal stability of spliced OmpX was examined via circular dichroism spectroscopy (CD) and compared to wildtype OmpX (b). Spliced OmpX unfolds similarly to wildtype OmpX, indicating a stable fold of the spliced version.

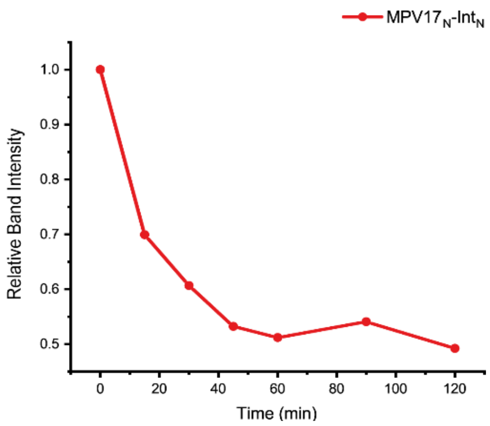

Supporting Information Figure 5. Reaction rate of MPV17 splicing. Relative SDS-PAGE band intensities of MPV17$_N$-Int$_N$ (Fig. 2) were determined and plotted against the ligation reaction time. In contrast to the analysis for OmpX (Fig. S2), values were calculated relative to time point zero instead of the educt, as the reaction rate was slower for this variant. Most of the conversion was achieved after 60 min. The yield after 120 min was determined to be at 50%.

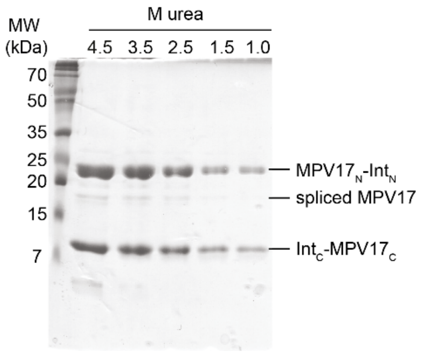

Supporting Information Figure 6. Purification of spliced MPV17. After the splicing reaction MPV17 could be further purified by diluting the splicing reaction in 6 M urea with aqueous buffer, in which MPV17 precipitated.

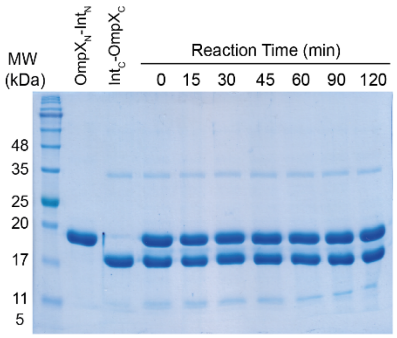

Supporting Information Figure 7. Intein splicing of OmpX in 0.5% sodium dodecyl sulfate (SDS). Intein splicing of OmpX in 0.5% SDS was conducted at 37 °C, otherwise following the same protocol as for splicing in 6 M urea. In contrast to splicing in urea product formation was not observed in SDS.

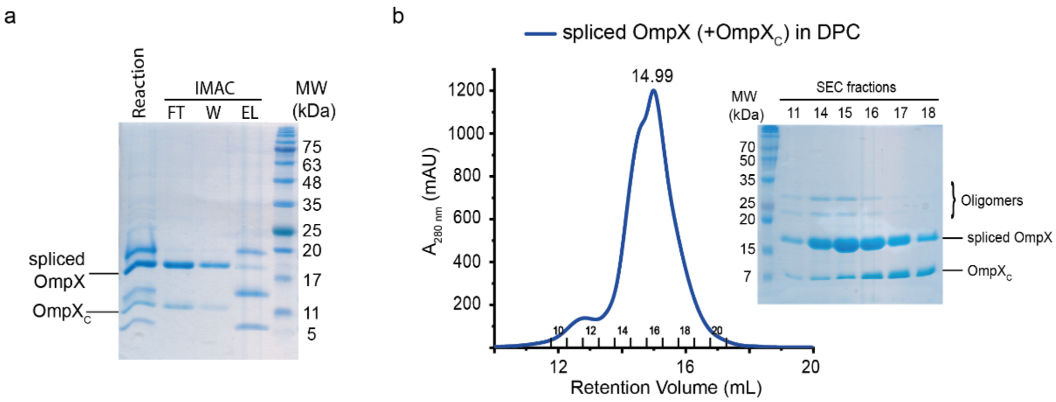

Supporting Information Figure 8. Purification of spliced OmpX and refolding. (a) SDS-PAGE of reversed immobilized metal affinity chromatography (IMAC) of the reaction mixture (left lane). Flow-through (FT) and wash fraction (W) contained spliced OmpX and $OmpX_C$ and were pooled for further purification and/or refolding. The elution fraction (EL) contained educts as well as the excised inteins Int_C and Int_N. (b) Size-exclusion chromatography (SEC) of in n-Dodecyl-phosphocholine (DPC) refolded spliced OmpX and fragment $OmpX_C$. SDS-PAGE indicates SEC fractions containing both spliced OmpX and $OmpX_C$, aswell as oligomeric species.

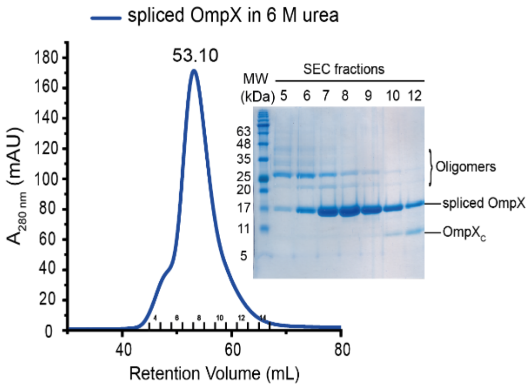

Supporting Information Figure 9. Separation of OmpX$_C$ from spliced OmpX via SEC. To remove the fragment OmpX$_C$ size-exclusion chromatography (SEC) in 6 M urea was carried out and clean fractions were pooled for subsequent experiments. Relevant SEC fractions are indicated in the adjacent SDS-PAGE.

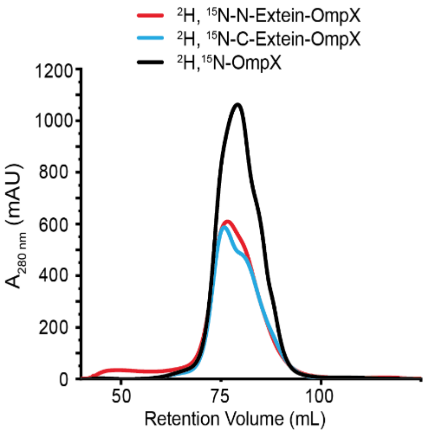

Supporting Information Figure 10. Size-exclusion chromatograms of (N-and C)-segmentally labeled OmpX and uniformly labeled OmpX in MSPΔH5 nanodiscs for NMR spectroscopy. The chromatograms each show a slight inhomogeneity towards higher retention volumes, as the samples also contain nanodiscs without incorporated protein of lower molecular weight.

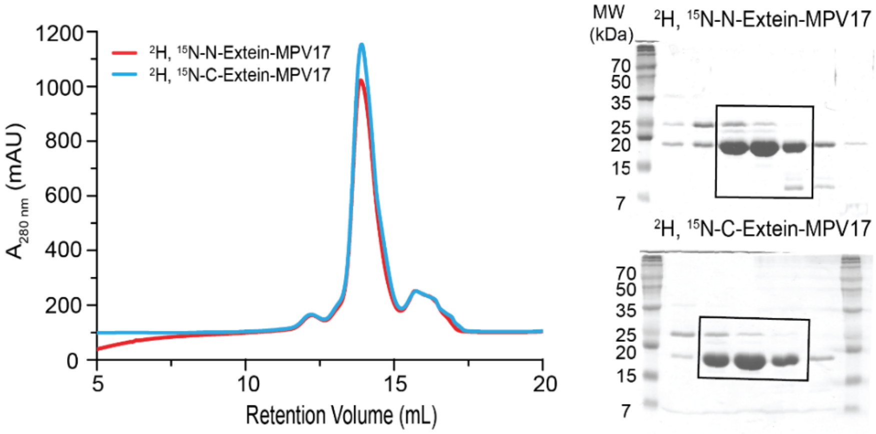

Supporting Information Figure 11. Size-exclusion chromatograms of (N-and C)-segmentally labeled MPV17 in detergent micelles. Respective SDS-PAGE gels are indicated adjacently. Indicated fractions were collected for NMR spectroscopy.

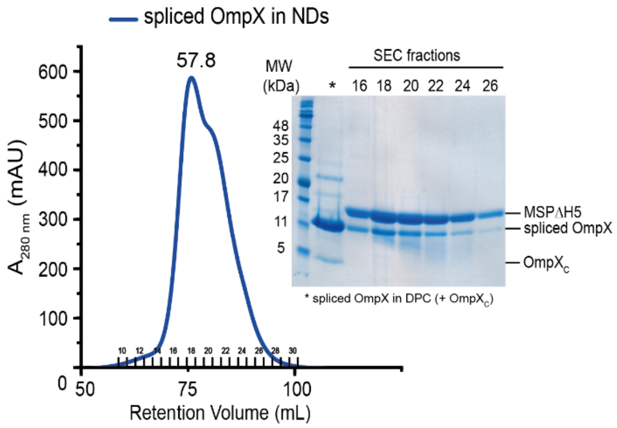

Supporting Information Figure 12. Separation of OmpX$_C$ from spliced OmpX by nanodisc insertion. To remove the fragment OmpX$_C$ nanodisc-assembly with subsequent size exclusion chromatography (SEC) was carried out. Relevant SEC fractions are indicated in the SDS-PAGE gel next to the chromatogram. As only correctly folded and monomeric protein can be assembled into the MSPΔH5 nanodiscs, with this step the unwanted side product could be easily removed. Spliced OmpX and OmpX$_C$ in n-Dodecyl-phosphocholine (DPC) before nanodisc assembly were also sampled for SDS-PAGE (indicated by *). The adjacent SEC fractions show that OmpX$_C$ could be removed effectively.

Supporting Information References

(1) Stevens, A. J.; Brown, Z. Z.; Shah, N. H.; Sekar, G.; Cowburn, D.; Muir, T. W. Design of a Split Intein with Exceptional Protein Splicing Activity. *J Am Chem Soc* **2016**, *138* (7), 2162-2165. DOI: 10.1021/jacs.5b13528.
(2) Sperl, L. E.; Hagn, F. NMR Structural and Biophysical Analysis of the Disease-Linked Inner Mitochondrial Membrane Protein MPV17. *J Mol Biol* **2021**, *433* (15), 167098. DOI: 10.1016/j.jmb.2021.167098.
(3) Hagn, F.; Etzkorn, M.; Raschle, T.; Wagner, G. Optimized phospholipid bilayer nanodiscs facilitate high-resolution structure determination of membrane proteins. *J Am Chem Soc* **2013**, *135* (5), 1919-1925. DOI: 10.1021/ja310901f From NLM Medline.
(4) Hagn, F.; Nasr, M. L.; Wagner, G. Assembly of phospholipid nanodiscs of controlled size for structural studies of membrane proteins by NMR. *Nat Protoc* **2018**, *13* (1), 79-98. DOI: 10.1038/nprot.2017.094.
(5) Fernandez, C.; Adeishvili, K.; Wüthrich, K. Transverse relaxation-optimized NMR spectroscopy with the outer membrane protein OmpX in dihexanoyl phosphatidylcholine micelles. *Proc Natl Acad Sci U S A* **2001**, *98* (5), 2358-2363. DOI: 10.1073/pnas.051629298.
(6) Schneider, C. A.; Rasband, W. S.; Eliceiri, K. W. NIH Image to ImageJ: 25 years of image analysis. *Nat Methods* **2012**, *9* (7), 671-675. DOI: 10.1038/nmeth.2089.
(7) Privalov, P. L. Stability of proteins: small globular proteins. *Adv Protein Chem* **1979**, *33*, 167-241. DOI: 10.1016/s0065-3233(08)60460-x.
(8) Lee, W.; Tonelli, M.; Markley, J. L. NMRFAM-SPARKY: enhanced software for biomolecular NMR spectroscopy. *Bioinformatics* **2015**, *31* (8), 1325-1327. DOI: 10.1093/bioinformatics/btu830.
(9) Hagn, F.; Klein, C.; Demmer, O.; Marchenko, N.; Vaseva, A.; Moll, U. M.; Kessler, H. BclxL changes conformation upon binding to wild-type but not mutant p53 DNA binding domain. *J Biol Chem* **2010**, *285* (5), 3439-3450. DOI: 10.1074/jbc.M109.065391.
(10) Hagn, F.; Wagner, G. Structure refinement and membrane positioning of selectively labeled OmpX in phospholipid nanodiscs. *J Biomol NMR* **2015**, *61* (3-4), 249-260. DOI: 10.1007/s10858-014-9883-6.
(11) Schwieters, C. D.; Kuszewski, J. J.; Tjandra, N.; Clore, G. M. The Xplor-NIH NMR molecular structure determination package. *J Magn Reson* **2003**, *160* (1), 65-73. DOI: 10.1016/s1090-7807(02)00014-9.
(12) Laskowski, R. A.; Rullmannn, J. A.; MacArthur, M. W.; Kaptein, R.; Thornton, J. M. AQUA and PROCHECK-NMR: programs for checking the quality of protein structures solved by NMR. *Journal of biomolecular NMR* **1996**, *8* (4), 477-486.
